# New Jersey State Committee of Arrangements

**Published:** 1892-09

**Authors:** Wm. Herbert Lowe

**Affiliations:** Resident State Secretary for New Jersey, U. S. V. M. A.; Paterson, N. J.


### UNITED STATES VETERINARY MEDICAL ASSOCIATION.

### NEW JERSEY.

*The New Jersey members are active, and have issued the following:*

DOCTOR . . . . . . . . . .

DEAR SIR:—It is my very pleasant duty to advise you of your appointment as a member of the New Jersey State Committee of

Arrangements—United States Veterinary Medical Association. A list of the members of this Committee is enclosed herewith, so that you may know who your associates are. It has been my object in the selection of this Committee to make it representative of the veterinary profession of New Jersey in the fullest sense of the term.

Dr. Hoskins writes me that the hall has been obtained and that the usual one and one-third rates have been conceded us by the Trunk Line Association, which covers our entire territory. A special effort is being made to make the forthcoming meeting at Boston a greater success than any preceding meeting of the Association, and the profession in New Jersey should be fully and ably represented.

It will be necessary to call a Committee meeting very soon in order to complete the local arrangements. Dr. Hoskins suggests that the Committee issue a circular setting forth the facts about the Boston meeting, to be sent to every veterinarian in our State. I trust you will assist the cause with your presence and counsel.

I should be very pleased to receive suggestions from you. Let me hear from you at once, before the Committee meeting is called.

Very respectfully,

Wm. Herbert Lowe,

*Resident State Secretary for New Jersey, U. S. V. M. A.*

---

New Jersey State Committee of Arrangements.

*Annual meeting at Boston, Mass., September 20th, 21st and 22d, 1892:*

Doctors William B. E. Miller, Camden; E. L. Loblein, New Brunswick; Richard R. Letts, Hoboken; Joseph Autenreith, Jersey City; D. J. Dixon, Hoboken; James D. Hopkins, Newark; James W. Hawk, Newark; E. R. Voorhees, Plainfield; J. Hueslen, Jr., Jersey City; A. T. Sellers, Camden.

Wm. Herbert Lowe,

*Resident State Secretary for New Jersey, U. S. V. M. A.*

Paterson, N. J., August 5th, 1892.

